# Re-orienting anti-malarial drug development to better serve pregnant women

**DOI:** 10.1186/s12936-022-04137-2

**Published:** 2022-04-12

**Authors:** Myriam El Gaaloul, Belen Tornesi, Flynn Lebus, David Reddy, Wiweka Kaszubska

**Affiliations:** 1 Medicines for Malaria Venture, Geneva, Switzerland; 2 FSG, Rue de Lausanne 82, Geneva, Switzerland

**Keywords:** Malaria in pregnancy, Equity in R&D, Inclusion of women, Antimalarial drugs, New medicines

## Abstract

Malaria is one of the most serious infectious diseases affecting predominantly low- and middle-income countries, where pregnant women are among the populations at risk. There are limited options to prevent or treat malaria in pregnancy, particularly in the first trimester, and existing ones may not work optimally in areas where the threat of drug resistance is rising. As malaria elimination is a key goal of the global health community, the inclusion of pregnant women in the adult population to protect from malaria will be key to achieving success. New, safe, and effective options are needed but it can take decades of evidence-gathering before a medicine is recommended for use in pregnancy. This is because pregnant women are typically not included in pre-registration clinical trials due to fear of causing harm. Data to support dosing and safety in pregnancy are subsequently collected in post-licensure studies. There have been growing calls in recent years that this practice needs to change, amplified by the COVID-19 pandemic and increasing public awareness that newly developed medicines generally cannot be administered to pregnant women from the onset. The development of new anti-malarials should ensure that data informing their use in pregnancy and breastfeeding are available earlier. To achieve this, a mindset change and a different approach to medications for pregnant women are needed. Changes in non-clinical, translational, and clinical approaches in the drug development pathway, in line with recent recommendations from the regulatory bodies are proposed in this Comment. The new approach applies to any malaria-endemic region, regardless of the type of *Plasmodium* responsible for malaria cases. By incorporating intentional and systematic data collection from pre-registration stages of development through post-licensure, it will be possible to inform on the benefit/risk balance of a new anti-malarial earlier and help ensure that the needs of pregnant individuals are addressed in a more timely and equitable manner in the future.

## Background

Malaria is an infectious disease predominantly of low- and middle-income countries. The latest World Health Organization (WHO) World Malaria Report [1] states that in 2020 there were an estimated 241 million cases of infection in 87 malaria endemic countries, resulting in 627,000 deaths, and more than a third of pregnant women in 33 African countries with moderate to high transmission had malaria. Data from regions outside of Africa are scarce, but more than 90 million pregnant women were estimated to be at risk of malaria in the Asia–Pacific region [2]. These are estimates and subject to change as malaria elimination progresses. For example, China was listed as a ‘malaria-endemic’ country with over 21 million pregnancies at risk [3], while the WHO has now certified the country as malaria-free [4]. The WHO envisages a 90% reduction in malaria incidence and mortality globally by 2030 compared with 2015 levels [5]. The inclusion of pregnant women in the adult population will be key to successful elimination campaigns as was recently highlighted in The Lancet Commission on Malaria Eradication [6].

Malaria poses specific risks both to pregnant women and the neonates. Firstly, altered immune response during pregnancy is thought to lead to increased susceptibility to infection [7]. Then, malaria in pregnancy (MiP) increases the risk of a severe form of the disease for the mother, and consequently severe anaemia [8]. Despite the increasing knowledge of the consequences of MiP [9], estimates of malaria-related maternal mortality in Africa are scarce and more studies are needed. However, there are numerous reports on the adverse effects of MiP on birth outcomes which include miscarriage, stillbirth, preterm birth, neonatal mortality, and babies born small for gestational age or with a low birthweight. It has been estimated, using meta-analysis, that 20% of all stillbirths in sub-Saharan Africa are attributed to *Plasmodium falciparum* malaria annually [10]. Malaria is also associated with a three–fourfold increased risk of miscarriage and preterm birth [11]. It has been estimated, using meta-analysis, that the prevalence of babies born small for gestational age is 32% [12]. According to the World Malaria Report [1], MiP resulted in an estimated 819,000 newborns with low birthweight in 33 African countries with moderate-to-high transmission in 2020. Low birthweight is linked to poor health outcomes and long-term morbidity risk [12]. Modelling suggests that prevention of malaria before conception or very early in pregnancy results in a greatly reduced incidence of low birthweight, especially in primigravidae [13]. Moreover, *Plasmodium vivax*, less prevalent globally but the dominant human malaria parasite in regions outside of Africa, also presents a major risk for pregnancy [14, 15].

The WHO recommends the following to control malaria in pregnancy: the use of an insecticide-treated bed nets (ITNs); effective case management with timely diagnosis and adequate treatment; intermittent preventive treatment using sulfadoxine-pyrimethamine (SP). Unless they sleep under an ITN, most women are not adequately protected in early pregnancy. This is because neither SP nor any of the six available artemisinin-based combination therapy (ACT) regimens, the standard of care in malaria, are recommended for use in first-trimester pregnancy. Quinine in combination with clindamycin is the WHO recommended treatment, but it is poorly tolerated [16]. If these drugs are not available or fail, only then an ACT or oral artesunate in combination with clindamycin is recommended [5]. Despite the WHO recommendation, ACT is being adopted by national guidelines as first-line treatment for first trimester pregnancy in some malaria-endemic countries [17]. Moreover, it is important to point out that the malaria epidemiological context outside of Africa poses unique challenges for pregnant women at risk of *P. vivax* malaria [2, 15]. The blood-stage of *P. vivax* infection can in most regions of the world still be treated with chloroquine, a drug that is considered safe in pregnancy [5]. The liver-stage of *P. vivax* infection is treated with primaquine and in some countries with the recently registered tafenoquine. Both drugs are recommended with glucose-6-phosphate dehydrogenase (G6PD) deficiency testing to assess the potential risk of haemolysis. However, G6PD status of the fetus cannot be determined antenatally in most malaria endemic settings [5, 15], hence pregnant women may be ineligible for these essential treatments to prevent *P. vivax* relapse.

To better address the needs of pregnant women, the malaria research community is working towards the following goals: (1) to generate evidence on the dosing and safety of currently available therapies; (2) to increase the options to prevent and treat malaria in pregnancy; and (3) to allow inclusion of pregnant women in community-wide approaches to eliminate and ultimately eradicate malaria. New tools especially in the first trimester of pregnancy and sustained dedicated funding are needed to support the realisation of these goals. This Comment, highlights the approaches that should be adopted to include pregnant women in the development of new anti-malarials. These proposals are based on the recommendations of the *Task Force on Research Specific to Pregnant Women and Lactating Women* [18] led by the National Institutes of Health, the *ConcePTION project* led by the Innovative Medicines Initiative [19] and the guidance provided by the regulatory bodies. Although this Comment does not focus on lactating women, historically they also have been excluded from clinical research in most disease areas. In the case of malaria, it has been suggested that most existing first-line treatments appear safe during lactation, however data remain limited, and more research is needed[20].

Safe and effective medicines for both prevention and treatment of malaria during pregnancy are urgently needed. The pipeline of new medicines in development must factor in the needs of pregnant individuals from the outset. From a research and development perspective, this requires the collection of supporting data and appropriate inclusion of pregnant and lactating women in the development of new medicines earlier than is currently practiced. The benefit/risk balance, underpinned by relevant data, will guide regulators, funders, healthcare professionals and patients to informed decisions in addressing the medical needs of all segments of the population at risk of malaria.

### Anti-malarials suitable for use in all stages of pregnancy are limited

The WHO defines a “*positive pregnancy experience*” as “*maintaining physical and sociocultural normality, maintaining a healthy pregnancy for mother and baby (including preventing or treating risks, illness and death), having an effective transition to positive labour and birth, and achieving positive motherhood (including maternal self-esteem, competence and autonomy)*” [21]. Current WHO guidelines for malaria recommend intermittent preventative treatment in pregnancy (IPTp) as part of antenatal care in the second and third trimesters, in combination with ITNs [5]. While multiple artemisinin-based combinations are recommended by the WHO from the second trimester for malaria treatment during pregnancy, for the first trimester quinine plus clindamycin are recommended. In pregnancy, the benefit/risk balance when taking any medicine must be considered from the perspective of both the mother and the child. Given the potential for harm, the safety requirements are more stringent than for non-pregnant adults and should be well defined by the relevant authorities. This explains partly the limited anti-malarial options for pregnant women today and the challenges in developing new ones.

### Treatment

A clear picture of the total burden of malaria in the early stages of pregnancy is lacking. However, malaria in the first trimester of pregnancy is associated with miscarriage, and the risk is higher for women with symptomatic and asymptomatic malaria (adjusted odds ratio 4·0 and 2·7, respectively) than for women who did not have malaria in the first trimester [11]. Women of reproductive age take ACT whilst there might be insufficient evidence on the safety of these medicines during the first trimester, should they become pregnant. Although non-clinical data have raised concerns [22], a meta-analysis of prospective observational studies concluded that compared to quinine, artemisinin treatment (mainly artemether-lumefantrine, AL) in the first trimester is not associated with an increased risk of miscarriage or stillbirth [23]. However, AL is not yet recommended by the WHO for first-trimester pregnancy despite being used for two decades. As the general risk of fetal malformations is high during first trimester pregnancy, a body of evidence is needed to support the safety claim for a new drug. Better alignment with the WHO, and other stakeholders, on what evidence is required is key to expediting access to medicines by pregnant women particularly in first trimester. Furthermore, beyond the first trimester optimization of the dosing regimen might be necessary to achieve equal efficacy to non-pregnant population as has been recently suggested for AL based on pharmacokinetic modelling [24].

### Intermittent preventative therapy

To date, 33 African countries have adopted IPTp to reduce the burden of MiP. In 2020, 57% of women visiting antenatal care clinics received at least one dose, but only 32% received three doses, well below the coverage target of 80% [1]. Pregnancies frequently occur in regions with a prevalence of parasite drug resistance markers to SP, the drug combination recommended for IPTp. So far, even in those areas, SP remains associated with reductions in low birthweight [25]. Although the precise relationship between the resistance markers and SP efficacy is not yet clear, this continues to be a cause for concern and the need for additional drug combinations for use in IPTp is pressing [26, 27]. Innovative approaches like monoclonal antibodies [28] and vaccines specific for pregnancy-associated malaria [29] might offer alternatives as they are generally considered lower risk options than chemical entities.

### Community-wide campaigns

In population-based elimination strategies, such as Mass Drug Administration (MDA) campaigns, a large proportion of the target population will consist of individuals with no clinical manifestations of acute malaria infection who will be exposed to the drug without gaining immediate benefit, analogous to population-wide immunisation campaigns for vaccine-preventable infectious diseases. Consequently, the safety threshold required is much higher than for treatment of symptomatic malaria infection. Pregnant women are currently excluded from such campaigns due to the more stringent benefit/risk balance that exists at the individual level for this population. Given that the success of elimination strategies may depend on the inclusion of pregnant women it will be essential to have a range of medicines considered safe in all stages of pregnancy [30]. Moreover, as asymptomatic malaria infection in pregnant women is associated with an increased likelihood of anaemia [31], there will be an added benefit to individuals participating in the elimination campaigns in averting this adverse consequence of untreated asymptomatic infection.

The need for additional antimalarial choices for pregnant individuals in first trimester of pregnancy, as well as the need to support drug resistance and malaria elimination efforts, are urgent. The research community has a major role to play in meeting these needs. It must expedite the availability of more drug options appropriate for use in all stages of pregnancy and systematically generate the necessary evidence throughout the discovery, development and post-licensure of new antimalarials to address this population.

### A paradigm shift towards the inclusion of pregnant women in clinical research

In recent years, there has been a growing awareness that the labelling of most medications does not contain sufficient information about the use in pregnancy [20, 32]. Historically, just 1.3% of pharmacokinetic (PK) trials registered from the 1960s to 2013 included pregnant women [33] and only 1% of pharmaceutical industry-sponsored Phase IV trials were designed specifically for pregnant women [34]. Of over 500 anti-malarial drug trials conducted between 1966 and December 2006, only 31 evaluated anti-malarials specifically in pregnant women and recommended dose regimens for pregnant women are all derived from studies in non-pregnant adults [35]. As pregnant women are not typically included during the development of medicines, data regarding safe use in pregnancy are collected in costly post-registration studies (e.g., pregnancy exposure registries, case–control studies and surveillance). In many malaria-endemic countries there are challenges to this approach due to the lack of robust pharmacovigilance systems, patients not being routinely followed-up, or limited resources. Therefore, it takes time to make efficacy and safety data of anti-malarials in pregnant population available via much needed meta-analyses and evidence synthesis [36, 37].

A paradigm shift towards the inclusion of pregnant women in clinical research is underway and necessary changes are starting to be proposed in some disease areas [38]. Several bodies have endorsed the shift, including the Council for International Organizations of Medical Sciences [39], the WHO [40], and regulatory agencies as reflected in updates of relevant guidance in recent years. In 2018, a Task Force on Research Specific to Pregnant Women and Lactating Women (PRGLAC) recommended to the US Congress the inclusion and integration of pregnant women and lactating women into the clinical research agenda [18] on the basis that the exclusion of these populations to-date has significantly limited scientific knowledge of therapeutic product safety, effectiveness and dosing. In line with these recommendations, the inclusion of pregnant women in clinical research should be extended to new anti-malarial drug development to accelerate access by this population to new medications in malaria-endemic countries. Consultations with the malaria community stakeholders, support from the pharmaceutical industry and funders will be important in implementing the new recommendations into practice.

### Proposed approaches to discovery and development of future anti-malarial drugs suitable for pregnant women

**Fig. 1:**
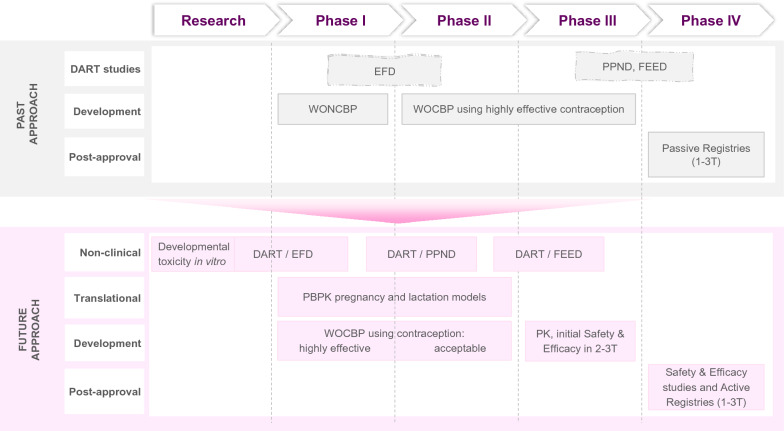
Proposed changes to the antimalarial research and development to better integrate the needs of pregnant individuals in the future. PAST APPROACH (top): Women of non-child-bearing potential (WONCBP) and women of child-bearing potential (WOCBP) with highly effective contraceptive use are included in clinical studies. The timing and extent of non-clinical Developmental and Reproductive Toxicity (DART) studies depends on the intended patient population. Generally, embryofoetal development (EFD) studies start in time to support clinical Phase II but pre-/postnatal development (PPND), as well as fertility and early-embryonic development (FEED) studies may be completed even post-licensure. Data on safety in pregnancy are collected passively in post-licensure registries. Pharmacokinetic (PK) data in pregnant and lactating women are rarely reported at the time of new medicine approval. FUTURE APPROACH (bottom): Lead compounds would be prioritized for progression to clinical development based on their non-teratogenic potential in in vitro assays, e.g., mammalian embryo exposure to compounds in a whole embryo culture (WEC) assay, or a zebrafish foetal development model. By performing the DART studies earlier and in relevant sequence, further selection of drugs for full development would be possible based on the risks identified in animal species. This would support earlier inclusion of WOCBP in clinical trials, with appropriate level of contraception [47], and pregnant women. Inadvertent pregnancies would be followed-up to assess maternal health, growth and development of the child. Utilising physiologically-based pharmacokinetic (PBPK) pregnancy or lactation models would inform on potential human foetal exposure or passage of the tested drug through the placenta and breastmilk, and hence support the justification for starting doses to be tested in clinical PK trials involving pregnant or lactating women. The PK trials would be initiated in parallel to the Phase III development for the general malaria population, once there is sufficient safety and efficacy evidence to derive an acceptable benefit/risk balance to start including pregnant or lactating women in the development of new drugs. Providing appropriate dosage and preliminary safety data in pregnant and lactating women in the first label of the registered medicine would expedite the commencement of further clinical studies and registries to fully characterize safety and efficacy. In particular, active pregnancy registries that capture inadvertent exposures in the first trimester, could help bridge the knowledge gap and provide some confidence to extend studies to this patient population

To tackle the urgent lack of anti-malarial options for prevention and treatment during all stages of pregnancy, there must be a commitment to systematically change the way the malaria research community approaches the discovery and development of new drugs. Adoption of the following practices is proposed (Fig. 1):

- Validation and routine use of predictive embryo and foetal development in vitro assays to efficiently screen and prioritize lead compounds based on their non-teratogenic profile before investing in more expensive Developmental and Reproductive Toxicity (DART) studies in animals. This early screening approach should not constitute an absolute no-go criterion where it might limit progress in addressing other critical unmet medical needs in malaria.
- Appropriate sequencing of DART studies to support the intentional inclusion of pregnant women in clinical studies during drug development. These studies evaluate the effects of potential drugs on one complete life cycle, from conception in one generation through the following generation. The timing and extent of DART studies is usually left to the discretion of the sponsor, depending on the target population [41]. Performing these studies earlier to use the results for decision-making would ensure that finite funding is channelled to clinical development of drugs most suitable for use during pregnancy.
- Utilising translational science approaches, such as physiologically-based pharmacokinetic (PBPK) models of pregnancy, to better anticipate how drug dosing might need to be adjusted for women in the second or third trimester, prior to including them in a clinical trial [42], or to semi-quantitatively assess the possibility of foetal exposure to the tested drug [43, 44]. Similarly, PBPK lactation models can be used to better anticipate the passage of anti-malarials into breast milk and inform on the need for a clinical study to assess safety for the newborn [45].
- Once compounds with appropriate non-clinical profile are progressed to clinical development, and there is sufficient safety and efficacy data in non-pregnant adults, simultaneous commencement of Phase I pharmacology trials for pregnant or lactating women parallel to Phase III trials for non-pregnant population.
- If the benefit/risk assessment is favourable, offering an option to women who inadvertently become pregnant while enrolled in a clinical trial to continue receiving treatment with appropriate follow-up including the newborn.

The overall aim of the above approach is to provide an initial data package that would inform on the benefit/risk balance for pregnant individuals at the time of new medicine registration for the general malaria population, and to support the collection of further evidence more expeditiously than currently practiced. Safety and efficacy interventional clinical trials in the third and second trimester of pregnancy and eventually in the first trimester should become routine practice. Active registries could help to capture data on the safety of inadvertent exposure to anti-malarials for the mother and the child, particularly in the first trimester.

To successfully adopt the drug research and development strategies described here, involvement of ethics committees [46], regulatory authorities, the WHO and normative bodies, investigators, patient communities and industry/development partners will be key. Such consultations would help determine what evidence is required to conduct clinical trials in pregnant women in pre-marketing setting; understanding that requirements for specific drugs might differ on case-by-case basis. Moreover, these consultations will expose existing barriers and explore ways to overcome them, including appropriate incentives to accelerate access to medicines by pregnant individuals as the proposed approach may require a greater upfront financial investment.

## Conclusions

Defeating malaria will not be possible without the intentional inclusion of pregnant and lactating individuals in clinical research. The malaria research community must prioritize the acceleration of discovery, development, and delivery of new, high-quality anti-malarial options for women of reproductive age who can and do become pregnant. In the near-term, data gathering on the safety and effectiveness of existing anti-malarials should continue. Furthermore, re-combining existing drugs to improve anti-malarial options appropriate for pregnant individuals and to make these more accessible, should be investigated. In the medium- to long-term, innovative strategies to identify new anti-malarial medicines that better serve the needs of pregnant women across all trimesters, as well as lactating women, must be explored. This can be achieved by adapting non-clinical and clinical approaches for the earlier inclusion of these patient populations in drug development research. Finally, the malaria elimination goals will be reached and lives saved from this preventable disease only by addressing the needs of the entire population at risk of malaria.

## Data Availability

Not applicable.

## Abbreviations

ACT: Artemisinin-based combination therapy
AL: Artemether-lumefantrine
DART: Developmental and reproductive toxicity
EFD: Embryofoetal development
FEED: Fertility and early-embryonic development
IPTp: Intermittent preventative treatment in pregnancy
ITN: Insecticide-treated net
MDA: Mass Drug Administration
MiP: Malaria in pregnancy
PBPK: Physiologically-based pharmacokinetic
PK: Pharmacokinetic
PPND: Pre-/post-natal development
PRGLAC: Task force on research specific to pregnant women and lactating women
SP: Sulfadoxine-pyrimethamine
WEC: Whole embryo culture
WOCBP: Women of child-bearing potential
WONCBP: Women of non-child-bearing potential
